# Blood pressure and heart failure

**DOI:** 10.1186/s40885-019-0132-x

**Published:** 2020-01-02

**Authors:** Gyu Chul Oh, Hyun-Jai Cho

**Affiliations:** Cardiovascular Center & Department of Internal Medicine, Seoul National University Hospital, 101 Daehak-ro, Jongno-gu, Seoul, South Korea

**Keywords:** Heart failure, Hypertension, Diastolic dysfunction, Left ventricular hypertrophy, J-curve

## Abstract

**Background:**

Hypertension is a leading cause of cardiovascular disease, stroke, and death. It affects a substantial proportion of the population worldwide, and remains underdiagnosed and undertreated.

**Body:**

Long-standing high blood pressure leads to left ventricular hypertrophy and diastolic dysfunction that cause an increase in myocardial rigidity, which renders the myocardium less compliant to changes in the preload, afterload, and sympathetic tone. Adequate blood pressure control must be achieved in patients with hypertension to prevent progression to overt heart failure. Controlling blood pressure is also important in patients with established heart failure, especially among those with preserved ejection fractions. However, aggressive blood pressure lowering can cause adverse outcomes, because a reverse J-curve association may exist between the blood pressure and the outcomes of patients with heart failure. Little robust evidence exists regarding the optimal blood pressure target for patients with heart failure, but a value near 130/80 mmHg seems to be adequate according to the current guidelines.

**Conclusion:**

Prospective studies are required to further investigate the optimal blood pressure target for patients with heart failure.

## Background

Hypertension (HTN) affects a substantial portion of the population worldwide, more than 7.5 million deaths per year are attributable to HTN [1], and HTN is the single most important contributor to mortality and morbidity. Individuals with high blood pressure (BP) are more susceptible to ischemic heart disease, and studies’ findings have shown that these patients may have a six-fold greater risk of a myocardial infarction [2]. HTN is also associated with the prevalence of atrial fibrillation [3] and ventricular arrhythmias [4]. Indeed, among those who participated in the Framingham Heart Study and not having histories of HTN and cardiovascular (CV) disease (*n* = 6859), the risk of developing CV disease in individuals with BPs ≥ 130/85 mmHg was almost triple that of participants with BPs < 120/80 mmHg [5]. In addition, diabetes and dyslipidemia frequently coexist with HTN [6, 7], which further increase the risk of CV disease.

The findings from different trials have demonstrated an association between long-standing HTN and heart failure (HF). A meta-analysis of 23 BP-lowering trials showed that 28.9% of the patients developed HF, which amounted to 8.5 events per 1000 patients [8]. Of the individuals in the Framingham Heart Study cohort, 91% of the participants with HF had a previous diagnosis of HTN, and compared with the normotensive individuals, the male and female patients with HTN had 2- and 3-fold increased risks, respectively, of developing HF [2]. Furthermore, the population-attributable risks of HF were 39 and 59% for the male and female participants, respectively [2], and the risk was even higher for the older individuals, with HF being attributable to elevated BP in up to 68% of the patients [9].

Chronic HTN causes structural and functional changes in the heart that ultimately lead to HF, which further increases mortality and morbidity. Although treating high BP intensively prevents and reverses myocardial changes in patients at risk of HF, defining the optimal BP target for patients with established HF is challenging, because the evidence is inconsistent and scarce. In this brief review, we aim to provide an insight into the pathophysiology of hypertensive heart disease and to summarize the current evidence relating to BP control in the prevention and treatment of HF.

### Pathophysiology of hypertensive heart disease

High BP increases the left ventricular (LV) afterload and peripheral vascular resistance, and prolonged exposure to an increased load leads to pressure- and volume-mediated LV structural remodeling [2, 10]. Ventricular hypertrophy is an initial compensatory mechanism in response to the chronic pressure overload that preserves the cardiac output and delays cardiac failure. However, the remodeled left ventricle is likely to decompensate, and HF can develop as a consequence of increased LV stiffness and the presence of diastolic dysfunction [11].

Diastolic dysfunction is one of the first changes observed in a heart that has been exposed to an increased load. In response to LV end-diastolic pressure elevations, the left ventricle becomes hypertrophied and more rigid, and additional changes are induced by neurohormonal pathways activated by the BP increase [12]. Ventricular hypertrophy can be divided into either concentric or eccentric hypertrophy by relative wall thickness (RWT). Concentric hypertrophy (RWT > 0.42) is associated with an increase in the thickness of the LV wall, while eccentric hypertrophy (RWT ≤0.42 is defined by the dilatation of the LV chamber [13]. A long-standing pressure overload is more likely to develop into concentric hypertrophy, while a volume overload is associated with eccentric hypertrophy. In chronic HTN, which involves both pressure and volume overloads, the LV hypertrophy can be concentric or eccentric, and these types are associated with HF with a preserved ejection fraction (HFpEF) and HF with a reduced ejection fraction (HFrEF), respectively. Demographic factors affect the response of the ventricle; hence, black patients are more likely than white patients to have concentric responses [14], and female and older patients are more prone to concentric changes [15, 16]. Patients with isolated systolic HTN and those with higher systolic BPs (SBPs) and ambulatory BPs are also more likely to exhibit concentric hypertrophy [17, 18]. Comorbid conditions can also affect the patterns of hypertrophy. For example, patients with diabetes are more prone to concentric changes [19], while patients with coronary artery disease and those who are obese are more likely to exhibit eccentric remodeling [20, 21]. Furthermore, the activation of neurohormonal pathways, including the renin-angiotensin system and the sympathetic nervous system [22], and extracellular matrix alterations induce changes in the ventricular mass and dimension [23].

Hypertensive HF primarily manifests as diastolic dysfunction, followed by concentric or eccentric LV hypertrophy. Diastolic dysfunction increases the LV filling pressure and left atrial (LA) volume, which, in turn, increase the pulmonary artery pressure [24].

The pathway from LV hypertrophy to overt HF is complex and unclear. Most patients with concentric hypertrophy develop HFpEF, but despite the absence of a history of myocardial infarction, some can progress to HFrEF. The development of HFpEF seems to be associated with changes in the extracellular matrix that cause progressive fibrosis of the myocardium and, subsequently, an increase in LV stiffness [25]. It is also possible that patients with eccentric LV hypertrophy will progress to either HFpEF or HFrEF. The changes in the heart caused by HTN were categorized over 25 years ago [12, 26], as follows: degree I: asymptomatic patients without LV hypertrophy, but with LV diastolic dysfunction; degree II: asymptomatic or mildly symptomatic patients with LV hypertrophy; degree III: clinical HF with a preserved ejection fraction (EF); and degree IV: dilated cardiomyopathy or HFrEF.

### Prognosis of patients with left ventricular hypertrophy

Cardiac structural changes are mostly caused by a chronic rise in the BP, and they are markers of preclinical or asymptomatic CV disease [27]. The presence of an LV strain pattern, that is, LV hypertrophy, on a 12-lead electrocardiogram is an independent predictor of the CV outcome [28]. The findings from two-dimensional echocardiography suggest that LV hypertrophy is a significant predictor of mortality [29]. The Framingham Heart Study’s findings showed that the incidence of CV events was significantly higher among the patients with an LV mass index (LVMI) > 125 g/m2 than that among the patients with normal LVMIs [30]. Ventricular hypertrophy is also a major predictor of stroke and renal outcomes [31]. Echocardiography can also detect diastolic dysfunction, and it provides information about chamber geometry and systolic function. Three-dimensional echocardiography and magnetic resonance imaging provide more reliable measurements of ventricular geometry and function, but less evidence is available regarding patients’ prognoses [32].

### Pulmonary edema

Patients with long-standing HTN are more sensitive to changes in pressure, volume, and sympathetic tone [33]. Although decompensated HF is usually considered a volume-overloaded state provoked by poor systolic function, the excess volume may not always be required for a patient to present with HF in those with LV hypertrophy and diastolic dysfunction. The reduced compliance of the ventricle and systemic vasculature in patients with hypertensive HF results in abnormal ventricular-vascular interactions [34]. The premature return of aortic pulse waves increases the resistance to the ventricular outflow, which, in turn, impedes the pulmonary venous flow towards the heart [33]. Consequently, small changes in the preload, afterload, or sympathetic tone can further increase the LV filling pressure, thereby disrupting the pulmonary capillary blood-gas barrier, which leads to flash pulmonary edema [12].

Hypertensive acute HF occurs when adverse conditions result in a volume redistribution and a shift from the splanchnic and peripheral vasculature to the pulmonary circulation. This ventricular-vascular uncoupling manifests as a rapid onset of pulmonary edema in patients with LV hypertrophy and diastolic dysfunction. Although intravenous diuretics are the first choice in treating acute HF with volume overload (Class I), reducing the preload and afterload using vasodilators (Class IIa, LOE B) should be also considered, as the volume overload might not be involved in the clinical congestion in some patients [33]. Residual pulmonary congestion persists in 30–40% of patients after hospital treatment [35], which suggests that relatively euvolemic patients require adequate diagnoses and treatment of pulmonary edema.

### Prevalence of hypertension among heart failure patients

The prevalence of HTN as an HF etiology varies geographically and temporally (Table 1). The findings from the Korean Heart Failure (KorHF) study, which recruited 3200 patients with HF from 2004 to 2009, showed that 36.7% of the patients had hypertensive HF [37]. More recently, the findings from the Korean Acute Heart Failure (KorAHF) study, which enrolled 5625 patients from 2011 to 2014, showed that only 4% of the patients had HF caused by HTN [36]. Up to 30% of the patients in the Acute Decompensated Heart Failure National Registry (ADHERE) had a hypertensive etiology, and this was more prevalent among the patients with normal EFs [44]. The results from studies of other large dedicated HF registries have shown that HTN was the primary cause of HF in 11–23% of the patients [45, 46].

**Table 1:** Comparison of dedicated heart failure registries

	KorAHF [36]	KorHF [37]	ASIAN-HF [38]	ATTEND [39]	ADHERE [40, 41]	ESC-HF-LT [42]
	2011–2014	2004–2009	2012–2015	2007–2011	2002–2004	2011–2013
N	5625	3200	5276	4837	159,168	4449
Age, years (SD)	68.5 (14.5)	67.6 (14.3)	59.6 (13.1)	73.0 (13.8)	72.4 (14.1)	69.4 (13.0)
Male, %	53.2	55.9	78.2	57.9	48.4	62.6
BMI, kg/m2	23.3 (3.9)	23.2 (4.0)	24.9 (5.1)			28.7 (5.4)
NYHA class III-IV, %	84.8	74.0	35	81.4	*76*	85.2
SBP, mmHg	131.2 (30.3)	130.5 (3.2)	118.4 (20.2)	145.5 (36.7)	143.9 (33.2)	133.5 (28.2)
DBP, mmHg	78.6 (18.8)	77.9 (18.0)	72.4 (12.6)	82.6 (22.6)	77.7 (20.2)	
HR, beats/minute	92.6 (26.0)	91.2 (25.4)	79.7 (16.2)	98.6 (29.1)		90.8 (25.3)
EF, %	37.7 ± 15.6	38.5 ± 15.7	28 [22, 33]	53.4% with EF <40%	37.8 ± 17.3	39.0 [30, 43]
Etiology (%)						
- Hypertension	4.0	36.7		18	Up to 30% in EF ≥ 55%	8.2
- Ischemic heart disease	37.6	52.3	47.0	31.1		56.5
Medical history (%)						
- Hypertension	62.2	46.5	51.9	69.3	73.9	65.6
- Ischemic heart disease	42.9		50.2		57.5	53.8
- Diabetes mellitus	40.0	30.5	40.4	36.2	*44*	39.0
- Atrial fibrillation / atrial flutter	28.5	20.8	17.9	39.6	30.9	44.0
- Renal insufficiency	14.3	9.2			30.1	25.3
- Stroke / transient ischemic attack	15.2	18.9	6.4	14.0		12.5
- Chronic lung disease	11.3	3.5	8.3	9.5	31.4	20.1
- Previous heart failure	47.8	29.6	64.1	36.1	75.6	70.9
Medications at discharge						
- ACE inhibitor	29.5	17.9	51.0	70–75	83.1	77.0
- Angiotensin receptor blocker	39.7	39.4	26.9			
- Beta blocker	49.9	58.6	78.8	67	80.1	72.6
- Aldosterone antagonist	44.9	53.1	59.4	40–50	32.8	53.9
- Diuretic		68.1	81.5		87.2	83.9

*SD* standard deviation, *BMI* body mass index, *NYHA* New York heart association functional class, *SBP* systolic blood pressure, *DBP* diastolic blood pressure, *EF* ejection fraction, *ACE* angiotensin converting enzyme

In contrast, the presence of HTN as a comorbid condition in patients with HF has become more pronounced over time. The findings from studies of the KorHF and KorAHF registries showed that the prevalence of HTN increased from 47 to 59% over 10 years [36, 37]. The findings from a study of the ADHERE showed that 69% of patients with HFrEFs and 77% of the patients with HFpEFs had elevated BPs [47]. The frequent coexistence of HTN and HF is observed across all regions. Indeed, 55.4% of the patients in the Asian Sudden Cardiac Death in Heart Failure registry [38] and 65.6% of the patients in the European Society of Cardiology Heart Failure Long-Term registry [42] were diagnosed with HTN and HF. Insurance claims data from the United States of America suggest that HTN was the most commonly co-occurring clinical condition among Medicare beneficiaries with HF [48].

Whether HTN is a cause or a contributor to the development of HF is not clear. Although a BP elevation alone may not be sufficient to trigger HF, it increases the risk of CV diseases progressing to HF. For example, the activation of neurohormonal pathways induced by a persistent BP elevation in addition to LV hypertrophy could lead to adverse modifications of postinfarct ventricular remodeling, rendering the heart vulnerable to the development of HF after a myocardial infarction [49]. Factors, including an increased afterload, reduced arterial compliance, and the lack of a response to vasodilators, also affect cardiac output in the context of HF [50].

### Blood pressure and heart failure prevention

Two issues arise regarding HF when treating HTN. The first is strictly controlling high BP to prevent structural remodeling and the development of HF. The presence of a J-curve association between BP and CV outcomes have been long debated, but evidence has been controversial [51, 52]. The current consensus is that strict control is mostly beneficial for hypertensive patients with low CV risk, while the risk of CV outcome increases in patients with high risk for coronary heart disease [53]. Recently, the findings from the Systolic Blood Pressure Intervention Trial (SPRINT), which assessed the role of intensive HTN treatment on a composite outcome that included HF, showed that a target SBP < 120 mmHg was associated with a 38% relative risk reduction in HF [54]. BP lowering in patients with ventricular hypertrophy can delay further remodeling and reduce the incidence of HF [55]. The LV hypertrophy induced by HTN is not unidirectional, and a regression of LV mass has been observed after the pharmacological treatment of elevated BP [43]. Improvements in LV hypertrophy have also been associated with reduced risks of CV events, including CV death, myocardial infarction, and stroke [56].

Identifying patients at an increased risk of developing hypertensive HF is important to enable attentive monitoring and begin timely treatment. LA enlargement in the absence of mitral valve disease could be a marker of diastolic dysfunction; this has been demonstrated by the correlation between the LA volume and the natriuretic peptide levels in asymptomatic patients with preserved systolic function [57]. Biomarkers such as uric acid, metalloproteinases, and natriuretic peptides, may also predict the development of HF in patients with HTN [58]. Patients with HTN and a high clinical risk of HF should be screened regularly for diastolic dysfunction or LV hypertrophy to prevent progression to advanced disease.

HTN treatment relies on many classes of drugs, including angiotensin-converting enzyme (ACE) inhibitors/angiotensin receptor blockers (ARBs), beta-blockers, calcium channel blockers (CCBs), and diuretics. A meta-analysis of randomized clinical trials that analyzed HF as an outcome showed significant reductions in the new-onset HF rates in association with all of these classes of drug, except for ARBs, which may have been a consequence of the small number trials reviewed. The findings from head-to-head comparisons of the different drug classes have shown that CCBs seem to be inferior at preventing HF compared with ACE inhibitors/ARBs, beta-blockers, and diuretics; however, the inferiority of the CCBs was largely attributable to differences in the use of concomitant drugs [59]. Thiazide-like diuretics, which are widely used to treat HTN, but are not frequently used in patients with HF, also reduced the new-onset HF rate compared with placebo [60], which suggests that reducing the BP itself is probably the most important factor in HF prevention. Indeed, for every 10mmHg reduction in SBP, the HF rate declines by 12% [54]. Currently, no specific class of drugs is recommended for the prevention of HF in patients with HTN, and patients should be treated according to the guidelines [61].

### Treatment of hypertension in patients with heart failure: the J-curve phenomenon

The other issue regarding HF and HTN is managing high BP in established HF patients. Treating high BP is more complicated in patients with established HF, but it remains important with respect to HF progression and patients’ prognoses. As all medications that have favorable effects on HF outcomes lower BP to some extent, we can assume that a close relationship exists between BP and HF outcomes. However, data describing the optimal BP in patients with HF are limited and contradictory. The findings from the OPTIMIZE-HF (Organized Program to Initiate Lifesaving Treatment in Hospitalized Patients with Heart Failure) trial suggested that BP elevations in patients with HF were associated with lower in-hospital mortality rates [46, 62]. A meta-analysis of 8000 patients with chronic HF also confirmed a trend towards better outcomes in patients with higher BPs [63]. Findings from the COPERNICUS (Carvedilol Prospective Randomized Cumulative Survival) and CHARM (Candesartan in Heart Failure: Assessment of Reduction in Mortality and Morbidity) trials showed that the benefits of these treatments persisted, regardless of a patient’s BP, but the statistical significance of the findings was marginal among the patients with lower BPs [64, 65]. Finally, the PARADIGM (Prospective Comparison of angiotensin receptor-neprilysin inhibitor (ARNI) with an angiotensin-converting enzyme (ACE) inhibitor to Determine Impact on Global Mortality and Morbidity in Heart Failure) study findings show that, although the benefit of sacubitril/valsartan over enalapril was still evident in patients with SBP < 120 mmHg, it was significantly more decreased than those with SBP ≥120 mmHg [66]. However, the findings of a recent prospective cohort study has demonstrated that a higher SBP, diastolic BP, and pulse pressure were associated with higher rates of adverse events among patients with incident HF [67].

**Fig. 1:**
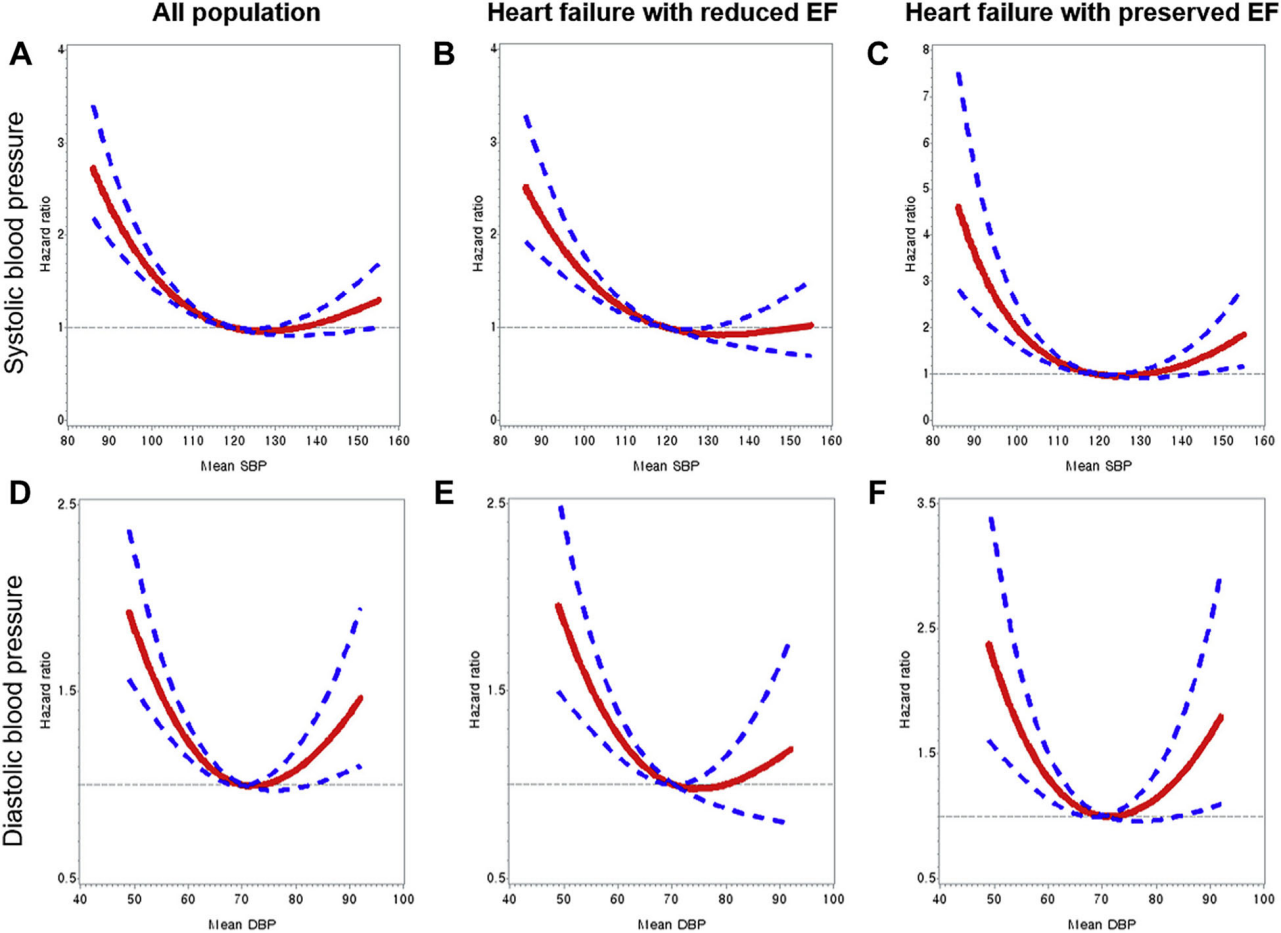
Restricted Cubic Splines Model for All-Cause Mortality According to On-Treatment BP. **a** SBP: all population. **b** SBP: heart failure with reduced ejection fraction (EF). **c** SBP: heart failure with preserved EF. **d** DBP: all population. **e** DBP: heart failure with reduced EF (EF). **f** DBP: heart failure with preserved EF. SBP, systolic blood pressure; DBP, diastolic blood pressure. Reprinted from JACC: Heart Failure, Vol 5, Lee SE, et al., Reverse J-Curve Relationship Between On-Treatment Blood Pressure and Mortality in Patients With Heart Failure, 810–819 No.11, 2017, with permission from Elsevier [36]

The relationship between BP and the HF prognosis is not necessarily always linear. A J-curve that is similar to that which describes the relationship between the BP and CV outcomes, has been indicated repeatedly in patients with HTN [68–70]. The findings from a study of the KorAHF registry showed that a reverse J-curve relationship was evident between the treatment of BP and the outcomes of patients who were hospitalized for HF [71], and that the risks of mortality and readmission increased at low and high BPs, with similar trends for patients with HFrEFs and HFpEFs (Fig. 1).

The trade-off between prescribing adequate doses of guideline-directed medical treatments and maintaining a lower BP threshold is an issue that many physicians encounter in daily practice. The benefits of treatment in relation to the outcomes must be weighed against the adverse effects induced by lower BPs. Although medications with survival benefits remain effective within lower BP thresholds, no definitive evidence is available that supports intensive BP treatment. The current evidence suggests that all patients with HF should receive triple therapy comprising ACE inhibitors or ARBs, beta-blockers, and diuretics, with the doses adjusted to maintain an adequate BP, and if a patient remains hypertensive, thiazide-like diuretics can be added [12]. Based on the available evidence, the Korean Society of Hypertension’s guidelines recommend an optimal BP that is close to 130/80 mmHg when treating patients with established HF [61].

## Conclusion

Among patients with chronic HTN, structural and functional changes in the heart can lead to the development of HF. BP management not only prevents asymptomatic HTN-mediated organ damage that can cause HF but can also prevent further disease progression. The intensive control of BP is recommended for patients with HTN who are at risk of HF; however, the optimal range within which BP should be controlled and the benefits of intensive BP control in patients with established HF remain unclear. Future randomized clinical trials are warranted to understand the complex association between BP and patients’ prognoses in the context of HF management.

## Data Availability

Not applicable.

## Abbreviations

ACE: Angiotensin-converting enzyme
ADHERE: Acute Decompensated Heart Failure National Registry
ARB: Angiotensin receptor blocker
BP: Blood pressure
CCB: Calcium channel blocker
CV: Cardiovascular
EF: Ejection fraction
HF: Heart failure
HFpEF: Heart failure with a preserved ejection fraction
HFrEF: Heart failure with a reduced ejection fraction
HTN: Hypertension
KorAHF: Korean Acute Heart Failure
KorHF: Korean Heart Failure
LA: Left atrial
LV: Left ventricular
LVMI: LV mass index
SBP: Systolic blood pressure
